# Estimation of age- and stage-specific Catalan breast cancer survival functions using US and Catalan survival data

**DOI:** 10.1186/1471-2407-9-98

**Published:** 2009-03-30

**Authors:** Ester Vilaprinyo, Montserrat Rué, Rafael Marcos-Gragera, Montserrat Martínez-Alonso

**Affiliations:** 1 Institut d'Investigació Biomèdica de Bellvitge (IDIBELL), Hospitalet de Llobregat, Catalonia, Spain; 2 Departament de Ciències Mèdiques Bàsiques, Facultat de Medicina, Universitat de Lleida-IRBLLEIDA, Lleida, Catalonia, Spain; 3 Unitat d'Epidemiologia i Registre de Càncer de Girona, Pla Director d'Oncologia, IdIBGi, Girona, Spain; 4 Departament d'Infermeria, Universitat de Girona, Girona, Spain

## Abstract

**Background:**

During the last part of the 1990s the chance of surviving breast cancer increased. Changes in survival functions reflect a mixture of effects. Both, the introduction of adjuvant treatments and early screening with mammography played a role in the decline in mortality. Evaluating the contribution of these interventions using mathematical models requires survival functions before and after their introduction. Furthermore, required survival functions may be different by age groups and are related to disease stage at diagnosis. Sometimes detailed information is not available, as was the case for the region of Catalonia (Spain). Then one may derive the functions using information from other geographical areas. This work presents the methodology used to estimate age- and stage-specific Catalan breast cancer survival functions from scarce Catalan survival data by adapting the age- and stage-specific US functions.

**Methods:**

Cubic splines were used to smooth data and obtain continuous hazard rate functions. After, we fitted a Poisson model to derive hazard ratios. The model included time as a covariate. Then the hazard ratios were applied to US survival functions detailed by age and stage to obtain Catalan estimations.

**Results:**

We started estimating the hazard ratios for Catalonia versus the USA before and after the introduction of screening. The hazard ratios were then multiplied by the age- and stage-specific breast cancer hazard rates from the USA to obtain the Catalan hazard rates. We also compared breast cancer survival in Catalonia and the USA in two time periods, before cancer control interventions (USA 1975–79, Catalonia 1980–89) and after (USA and Catalonia 1990–2001). Survival in Catalonia in the 1980–89 period was worse than in the USA during 1975–79, but the differences disappeared in 1990–2001.

**Conclusion:**

Our results suggest that access to better treatments and quality of care contributed to large improvements in survival in Catalonia. On the other hand, we obtained detailed breast cancer survival functions that will be used for modeling the effect of screening and adjuvant treatments in Catalonia.

## Background

Mortality from breast cancer has been decreasing in the majority of industrialized countries since the beginning of the 1990s. Extended use of mammography screening and improvements in adjuvant therapies have been indicated as the causes of this decline. Adjuvant therapies are drugs, usually chemotherapy or hormonotherapy, used as additional treatments for patients with cancers that are thought to have spread beyond their original sites.

On one hand, screening with mammography may reduce breast cancer mortality, because more tumors are detected at earlier stages where survival time is longer. On the other hand, concurrently with mammography use, the introduction of adjuvant therapies also may have increased the survival time at all stages of the disease and modified the survival functions.

Based on a collaborative effort, seven research groups modeled the US breast cancer mortality trends from 1975 to 2000. The Cancer Intervention and Surveillance Modeling Network (CISNET) is a consortium of the US National Cancer Institute that sponsored and oversaw this initiative [1]. All groups shared the same model inputs: incidence, survival after breast cancer diagnosis and mortality by other causes of death, over time. The goal of the modeling process was to determine the contribution of screening mammography and adjuvant treatments on the reduction in breast cancer mortality [2]. One of the CISNET groups, lead by Marvin Zelen and Sandra Lee at the Dana-Farber Cancer Institute in Boston [3], developed a stochastic model that requires specific breast cancer survival probability density functions (*pdf *s) by age and disease stage at diagnosis.

Catalonia is an autonomous region of Spain with authority over health-care planning, administration, and provision since 1985. It has approximately 7.2 million inhabitants, one sixth of the Spanish population. The Catalan Health Department has implemented an independent information system and preventive programs in the region. Population breast cancer screening programs were initiated in the early 1990s [4]. All this facilitates the collection of almost all the required inputs to use the Lee and Zelen's model to study the impact of mammography and adjuvant treatments in Catalonia. But Catalan survival information is not reliable enough to directly derive the survival functions by age and disease stage at diagnosis. While stage-specific Catalan survival functions are robust, when stratifying by age and stage the number of women at risk over time decreases and the survival estimates are unstable (see Additional file 1). Since younger women are affected by more agressive tumors, it is important to estimate age- and stage-specific survival functions.

This work presents the methodology used to estimate age- and stage-specific Catalan breast cancer survival functions from scarce Catalan survival data by adapting the age- and stage-specific US functions. In addition, we compared breast cancer survival in Catalonia and the USA in two time periods, before and after the introduction of screening mammography.

## Methods

This section is structured in four parts: a) The obtention of the US breast cancer hazard rate functions, by stage and also by age and stage; b) The description of the existent and inferred Catalan survival data; c) The estimation of the hazard ratios (HR) or relative hazards that compare Catalonia versus the US, which sometimes are time-dependent; and d) The estimation of age- and stage-specific breast cancer survival functions for Catalonia using the US functions and the estimated HR.

### US survival data

#### Background survival data

To understand how early screening and adjuvant therapies contributed to the reduction of the breast cancer mortality it is necessary to consider breast cancer survival prior to these two interventions. During the period of 1975–79, the two factors were absent in the USA. Thus, *background *data and functions refer to these years.

Survival information following cancer diagnosis was retrieved from the National Cancer Institute's Surveillance Epidemiology and End Results (SEER) Program [5]. SEER currently collects and publishes cancer incidence and survival data from population-based cancer registries covering approximately 26% of the US population.

For the period 1975–79, women diagnosed with breast cancer were classified using the SEER historical summary of disease stage at diagnosis (localized, regional, and distant). Localized is defined as disease confined entirely to the organ of origin, regional is disease that has extended beyond the limits of the organ of origin into surrounding organs or tissue and/or regional lymph nodes, and distant is disease with metastasis. This classification was used in the comparison of survival in the USA and Catalonia. From the historical summary stage information and additional data on the extent of disease, the CISNET inferred breast cancer survival functions for the US population in 5 age groups (30–39, 40–49, 50–59, 60–69 and 70–84 years) and for 5 disease stages at diagnosis (American Joint Committee on Cancer (AJCC) staging system: I, II-node negative (II-), II-node positive (II+), III, IV) [6]. These 25 age- and stage-specific functions will be used to obtain the detailed Catalan survival functions.

#### Recent survival data

Estimates of the US breast cancer specific survival functions (hazard, density and cumulative survival) for the period 1990–2001 were obtained using data output from the SEER*Stat Survival software developed at the National Cancer Institute [5]. The AJCC disease stage information is provided in detail for this period: I, II, III, IV, and whether regional nodes are affected or not. Data selection was done using the default parameters and the *Display Standard Life by Cause-Specific Survival *option. Women aged 30–84 years and with a diagnosis of breast cancer were grouped in stages I, II-, II+, III and IV.

#### Hazard functions

The previously mentioned SEER and CISNET data sources record cases with 25 years of follow-up after diagnosis for women diagnosed during the *background *period, and cases with 16 years of follow-up for women diagnosed during the *recent *period. The information provided was the number of women alive at the beginning of the interval (*N*_*t*_) and the number of breast cancer deaths (*D*_*t*_) and women lost to follow-up (or withdrawals, *W*_*t*_) in time intervals of 1 year [*t*, *t *+ 1). Then the conditional hazard rates () by age and stage were estimated as the *number of breast cancer deaths *divided by *person-years at risk * = *N*_*t *_- 0.5(*D*_*t *_+ *W*_*t*_):(1)

The hazard rates estimated using expression (1) draw a step function with fluctuations. We smoothed these (*u*) and obtained continuous hazard rate functions using a restricted cubic spline model (natural splines) [7]. First, we used the function rc_spline from the STATA software to create a series of covariates that are functions of the independent variable *time from diagnosis *and predefined knots [8-10]. These covariates take the form of piecewise cubic polynomials between adjacent knots. Then, we run a weighted linear regression model with dependent variable (*u*) estimated in (1) and the specified covariates. We used knots at the 1.5, 2.5, 4.5, 8.5, 14.5 and 20.5 time points for period 1975–79, and at 1.5, 2.5, 4,5 and 8.5 for period 1990–2001. We used  as frequency weights to account for the precision of (*u*).

We assumed that the number of breast cancer deaths in a specific time interval [*t*, *t *+ 1) follows a Poisson distribution with parameter (*t*), being (*t*) the smoothed hazard rate in the midpoint of the interval. Therefore, (*t*) is the expected number of deaths, *E*_*t*_, in the time interval [*t*, *t *+ 1). To estimate the variance and point-wise 95% confidence intervals of the US hazard rates we used a bootstrap approach based on resampling the Pearson residuals , being *O*_*t *_the observed number of breast cancer deaths in each time interval (see Appendix for further details).

We derived three groups of US hazard functions:

1. For period 1975–79, 3 stage-specific functions, using the historical stage classification (localized, regional, and distant).

2. For period 1990–2001, 5 stage-specific functions using the AJCC staging classification (I, II-, II+, III, IV).

3. For period 1975–79, 25 age- and stage-specific functions using age groups (30–39, 40–49, 50–59, 60–69, 70–84 years) and the AJCC staging classification (I, II-, II+, III, IV).

### Catalan survival data

#### Background and recent survival data

Catalan breast cancer survival data is not available at the population level. There are two population based cancer registries that cover an area of 20% of the Catalan population. We obtained breast cancer survival data from one of these registries, the *Girona Cancer Registry *(GCR), which currently covers an area of 700 000 inhabitants and represents approximately 10% of the Catalan population. Breast cancer data from 1975 to 2005 collected by the GCR was considered representative of the Catalan breast cancer data. In 1986 the female population covered by the registry was 227 228 women, 7.45% of the Catalan female population (3 050 749 women) [11]. The reporting system was based on information from the area hospital records, pathology laboratories, and death certificates extracted from the Catalan Mortality Registry of the Catalan Government's Department of Health [12]. For the period 1980–89, the estimated exhaustivity was 96.7%, 93.2% of cases were histologically verified [11], and the percentage of unstaged cases was around 7%. For comparison, SEER provided 4% of non-classified stages for the same period.

For *background *analysis we used the period 1980–89 for two reasons. First, during this period screening and adjuvant therapies had no impact on the Catalan population. And second, data prior to this period was incomplete and inaccurate. For the *recent *analysis, all women incident from 1990 to 2001 were included. Ages selected for the analysis were 30 to 84 years old. Additional file 1 presents the number of women diagnosed of breast cancer by age and stage in the GCR.

#### Relative survival

Relative breast cancer survival is defined as the ratio between observed breast cancer survival and expected survival in the general population. It corrects the excess of mortality experienced by patients diagnosed with breast cancer. Relative survival can be interpreted as the probability of cancer survival after adjustment for competing causes of death [13] and is a close estimate of cause-specific survival [14]. Relative survival was estimated using a web-based application (WAERS [15]) developed by the Catalan Institute of Oncology which uses the Hakulinen's method to estimate the expected survival [16], the Kaplan-Meier's method to estimate the observed survival and the Greenwood's method [17] to estimate confidence intervals.

WAERS provided the relative cumulative survival from GCR data. Then relative breast cancer cumulative survival data were smoothed using the same method as for US hazards. We fitted a cubic spline (knots at the 1.5, 2.5, 4.5, 8.5, 10.5 and 12.5 time points) and a regression model weighted by the number of women at risk at the beginning of each interval.

### Hazard ratios (HR) of breast cancer mortality, Catalonia versus USA

Our goal was to obtain age- and stage-specific Catalan breast cancer survival functions. But the number of breast cancer cases in the GCR precluded estimation of these functions from the GCR data. Thus, we looked for a relation between the USA and the GCR (*t*) at the disease stage level, the hazard ratio (HR).

Since the size of the US population covered by SEER and the Girona province populations were very dissimilar, we used the USA data as reference data. First, we used the GCR *relative cumulative survival *and the *number of all-cause deaths *to estimate the *number of deaths due to breast cancer *(*D*^*bc*^) in the GCR at each year interval [*t*, *t *+ 1) after diagnosis (see first section in the Appendix for further details).

Second, we multiplied the  by the GCR *person-years at risk *to estimate the *expected number of breast cancer deaths *(*expected*^*bc*^) in the GCR, if the hazard rates were those of the USA (see second section in the Appendix). Third, we fitted a Poisson regression model with dependent variable  and with the  as an *offset *variable. This corresponds to a proportional hazards model, with the usual Cox baseline hazard *λ*_0 _replaced by the known hazard function from the US data. When the proportional hazards assumption was not fulfilled we included a function with time from diagnosis as a covariate. The *log *of time (*log*(*t*)) was the function that worked best. We included it in the models when it was statistically significant [18].

We checked for overdispersion by fitting negative binomial regression models and assessing the significance of the extra variation parameter. The Poisson model was adequate in all cases. In addition, we assessed the goodness-of-fit of the model using the deviance. All the final models had non-significant p-values (> 0.05) for the deviance *χ*^2 ^test. Degrees of freedom were equal to the number of observations minus 1, or minus 2 if time was included as a covariate.

The following equations reflect the Poisson model and the expression used to obtain the estimated HR:(2)

Then, the stage-specific hazard ratios were obtained as:(3)

The model was fitted for the first 14 years after breast cancer diagnosis, because after 14 years of follow-up the number of breast cancer deaths approaches 0. After year 14 we assumed that the HR was constant and equal to the year 14 estimated value.

Ninety-five percent confidence intervals (95% CI) for the HR were obtained using the expression:

(4) *exp*(*log*(*HR*) ± 1.96 *Var*(*log*(*HR*))^1/2^)

When the HR was constant over time (*log*(*HR*) = *β*_0_), the 95% CI for the HR were estimated as:

(5) *exp*(*β*_0 _± 1.96 *Var*(*β*_0_)^1/2^)

When the HR was time-dependent (*HR*(*t*)), the variance of the *log*(*HR*(*t*)) was obtained using the expression:

(6) *Var*(*log*(*HR*(*t*))) = *Var*(*β*_0_) + *log*(*t*)^2 ^*Var*(*β*_1_) + 2 *log*(*t*) *Cov*(*β*_0_, *β*_1_)

Then, we used expression (4) to obtain the 95% CI of the HR.

For the period of time prior to the dissemination of mammography (*background*), we used the 1975–1979 US hazard rates and the 1980–1989 GCR person-years at risk to estimate the GCR breast cancer deaths (*expected*^*bc*^). We performed this analysis by historical stage of disease (localized, regional, distant). For the *recent *period, we used the 1990–2001 data from the GCR and the 1975–79 data for the USA, using the AJCC five disease stages at diagnosis (I, II-, II+, III, IV).

The following scenarios provided the periods chosen for the comparisons:

• **C1**: Catalonia 1980–89 *vs *the USA 1975–79

• **C2**: Catalonia 1990–2001 *vs *the USA 1975–79

• **C3**: Catalonia 1990–2001 *vs *the USA 1990–2001

Comparisons **C1 **and **C2 **provide the HR needed to perform estimations of the Catalan breast cancer age-and stage-specific *λ*(*t*) functions for *background *and for *recent *time periods by using  (*t*) in 1975–79.

Additionally, from the HR obtained in **C1 **and **C3**, we can assess the differences in breast cancer survival between the USA and Catalonia, in the recent past, before the dissemination of screening with mammography.

### Estimation of age and stage-specific Catalan survival functions: *λ*, cumulative survival, and pdfs

We used the age- and stage-specific USA  functions in the period 1975–79 and the HR by stage from **C1 **and **C2 **to estimate the age- and stage-specific Catalan  functions for the periods 1980–89 and 1990–2001, respectively. We used the same HR for all age groups within disease stages:(7)

To obtain the AJCC (*t*) for Catalonia in 1980–89 we applied the historical stage *localized *HR to the USA hazard rate functions for AJCC stages I and II-. We used the historical stage *regional *HR for AJCC stages II+ and III, and the *disseminated *HR for the AJCC stage IV.

We also assessed whether the (*t*) functions, estimated using expression (7), fit the data well by using the goodness-of-fit deviance statistic. We obtained the deviance between the estimated breast cancer deaths in the GCR and the predicted number of deaths obtained assuming a Poisson distribution with parameter (*t*) with the offset term being the number of *person-years at risk*. In all cases the p-values of the *χ*^2 ^deviance tests were higher than 0.01, except for C1-Disseminated, C2-I, C2-II+, and C3-II+.

To estimate confidence bands for the variance of  we used the delta method [19]. First, we applied the logarithmic transformation:(8)

Then, assuming independence of the hazard ratios and the USA hazard rates:(9)

The term  was obtained from the Poisson regression models and the term  was obtained using the delta method as follows. The delta method stays that if we want to approximate the variance of *G*(*X*) where *X *is a random variable with mean *μ *and *G *is differentiable, then

(10) *G*(*X*) = *G*(*μ*) + (*X *- *μ*)*G' *(*μ*)

and

(11) *Var*(*G*(*X*)) = *Var*(*X*) * [*G' *(*μ*)]^2 ^

Therefore,(12)

with  being the variance obtained by bootstrap as described in subsection *Hazard functions *of the *Methods *section.

Cumulative survival is related to the hazard rate through . Thus, the cumulative survival function in Catalonia can be estimated as:(13)

Once the cumulative survival function *S*(*t*) is estimated, the survival  can be obtained from the relation [17]:

(14) *pdf *(*t*) = -*dS*(*t*)/*dt*

### Software

We used the Mathematica v6.0.3 Grid Edition software package for almost all computations [20]. STATA 10.0 was used to smooth data, and to fit the Poisson models [21]. SEER*Stat provided USA breast cancer specific cumulative survival for period 1990–2001 [5].

## Results

### USA hazard functions

**Figure 1:**
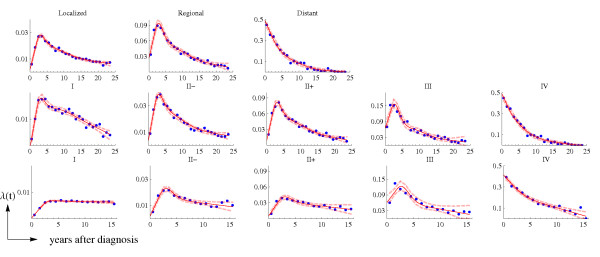
**Stage-specific US hazard rates: observed (dots) and the smoothed function (continuous line)**. The upper row corresponds to the hazard rates () for the SEER historical stage, period 1975–79 (**C1**); the middle row corresponds to  for the AJCC stages, period 1975–79 (**C2**); and the bottom row corresponds to  for the AJCC stages, period 1990–2001 (**C3**). Dashed lines correspond to the 95% point-wise confidence intervals.

**Figure 2:**
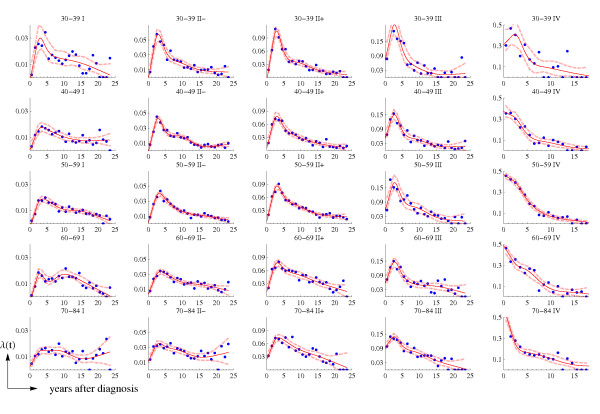
**Age- and stage-specific US hazard rates in 1975–79: observed (dots) and the smoothed function (continuous line)**. Dashed lines correspond to the 95% point-wise confidence intervals. Disease stage classified according to the AJCC staging system.

Figure 1 shows the raw and smoothed US hazard rates, by stage and time period (historical and AJCC stages for 1975–79 and AJCC stages for 1990–2001). Figure 2 presents the observed and smoothed US hazard rates, by age groups and AJCC stages, for the 1975–79 (*background*) period. The hazard rate functions in Figure 2, together with the estimated HR, will be used to derive the hazard rate functions in Catalonia for the background (1980–89) and recent (1990–2001) time periods.

Data in Figure 2 displays the high variability of the observed US hazard rates for young women at favorable stages and justifies the need for smoothing and deriving these functions for Catalonia.

### Catalan versus the US hazard ratios

Table 1 presents the coefficients and standard errors of the Poisson models that make it possible to estimate the HR. When the HR are not time-dependent (*β*_1 _= 0) the estimated HR are *log*(*β*_0_). In three situations both parameters *β*_0 _and *β*_1 _were not statistically different from 0, meaning that the compared (*t*) functions for Catalonia and the USA were similar.

**Table 1:** Fitted Poisson models that compare the hazard rate functions of the USA and Catalonia (Cat).

Comparison	Stage	*β*_0_		*β*_1_		Cov(*β*_0_, *β*_1_)
		Coef.	SE	Coef.	SE	
Cat 1980–89 vs USA 1975–79	Localized	0.5511	0.0786	-	-	-
	Regional	0.2392	0.0938	0.1797	0.0610	-0.0048
	Distant	0.2667	0.0898	-	-	-

Cat 1990–2001 vs USA 1975–79	I	-2.9278	0.9619	1.0937	0.5068	-0.4706
	II-	-1.1482	0.4722	0.5589	0.2776	-0.1223
	II+	-0.8711	0.2304	0.3902	0.1417	-0.0299
	III	-	-	-	-	-
	IV	-0.4499	0.1313	0.3840	0.1125	-0.0092

Cat 1990–2001 vs USA 1990–2001	I	-	-	-	-	-
	II-	-0.5765	0.4614	0.5387	0.2703	-0.1160
	II+	-0.1054	0.2240	0.2837	0.1370	-0.0279
	III	-	-	-	-	-
	IV	-0.2916	0.1300	0.3127	0.1095	-0.0087

Note: The hazard ratios are obtained using the expression . When coefficients are not reported ("--") means that they were not statistically significant. Catalan data refer to the Girona Cancer Registry data.

**Figure 3:**
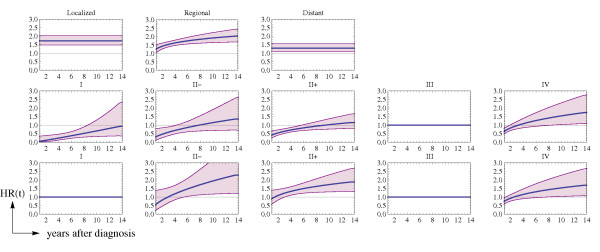
**Estimated hazard ratio functions, Catalonia versus the USA**. The upper row corresponds to the  for comparison C1: Cat 1980–89 versus the USA 1975–79; the middle row corresponds to C2: Cat 1990–2001 versus the USA 1975–79; and the bottom row corresponds to  C3: Cat 1990–2001 versus the USA 1990–2001. Dashed lines correspond to the 95% point-wise confidence intervals.

Figure 3 shows the estimated HR and their 95% confidence intervals. Horizontal lines that correspond to HR = 1 refer to non statistically different  functions. HR > 1 indicate that the risk of breast cancer death in Catalonia was higher than in the USA and HR < 1 mean the opposite. It is interesting to note that in some cases the HR values cross the *HR *= 1 line, indicating a change in the risk relation which needs to be interpreted by taking into account the confidence intervals. In addition, when the  are low, changes in the HR may not be rellevant, for example in the early stages of disease.

The upper row of Figure 3 compares Catalonia 1980–89 with the USA 1975–79 (**C1**). For the three historical stages, the hazard rates in Catalonia were higher than in the USA. For the regional stage, HR increased with time, indicating an increase in mortality risk over time in Catalonia in relation to the USA.

The middle row of Figure 3 compares Catalonia 1990–2001 with the USA 1975–79 (**C2**). For stages I, II- and II+ the 1990–2001  were lower than the 1975–79 , specially during the first years after diagnosis. For tumors with more advanced stages there were not statistically significant differences in the risk of death. Considering the comparisons (**C1**) and (**C2**), in Catalonia during the 1990s there was an important improvement in breast cancer survival.

The lower row of Figure 3 compares Catalonia and the USA in the same time period, 1990–2001 (**C3**). The (*t*) functions for AJCC stages I and III, in the USA and Catalonia, were similar. For stages II-, II+ and IV the  and  were similar for the first years after breast cancer diagnosis, but there was a tendency towards an increased risk of death in Catalonia that is statistically significant for later years.

**Figure 4:**
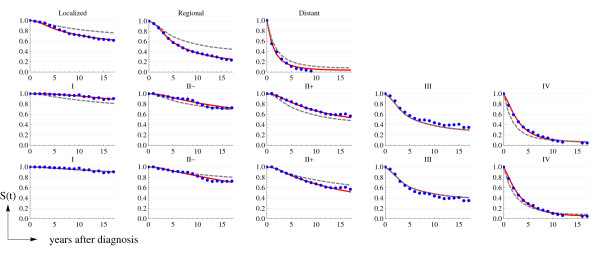
**Cumulative survival functions**. The upper row corresponds to C1: Cat 1980–89 versus the USA 1975–79; the middle row corresponds to C2: Cat 1990–2001 versus the USA 1975–79; and the bottom row corresponds to C3 Cat 1990–2001 versus the USA 1990–2001. Observed Catalan data (dots), estimated Catalan functions using the  and Catalan versus the US  (continuous line), and the US cumulative survival function (dashed line).

Figure 4 presents the cumulative survival functions for the three comparisons **C1**, **C2 **and **C3**. Each graph presents a) the observed Catalan cumulative survival obtained from the GCR data (dots), b) the US cumulative survival, and c) the estimated Catalan cumulative survival obtained by multiplying the corresponding HR by the US hazard rate functions. Figure 4 is consistent with Figure 3 and allows a more intuitive interpretation of differences in breast cancer survival in the USA and Catalonia before and after the utilization of screening mammography (upper and bottom rows). For instance, cumulative survival after 5 and 10 years from diagnosis is higher for all stages in the USA than in Catalonia for the *background *period, while lines corresponding to period 1990–2001 are very similar for both populations (see also Table 2).

**Table 2:** Estimated relative cumulative survival at five (*S*(5)) and ten (*S *(10)) years after breast cancer diagnosis in Catalonia and the USA.

Source	Stage	*N*_5_	*S*(5)	IC(0.95)	*N*_10_	*S*(10)	IC(0.95)
Cat 1980–89	SEER Historical stage						
	Localized	433	0.87	0.84 – 0.91	312	0.71	0.66 – 0.77
	Regional	384	0.58	0.54 – 0.62	207	0.38	0.34 – 0.42
	Distant	23	0.11	0.07 – 0.18	2	-	-
USA 1975–79							
	Localized	16198	0.9	0.90 – 0.91	12847	0.82	0.82 – 0.83
	Regional	11056	0.7	0.69 – 0.70	7172	0.54	0.54 – 0.55
	Distant	603	0.2	0.18 – 0.22	219	0.11	0.10 – 0.12

Cat 1990–2001	AJCC stage						
	I	389	0.99	0.96 – 1.01	171	0.98	0.94 – 1.02
	II-	195	0.92	0.87 – 0.97	86	0.82	0.75 – 0.90
	II+	309	0.84	0.80 – 0.89	138	0.67	0.62 – 0.73
	III	103	0.58	0.50 – 0.66	41	0.44	0.36 – 0.54
	IV	36	0.29	0.21 – 0.40	10	0.1	0.05 – 0.19
USA 1990–2001							
	I	103291	0.98	0.97 – 0.98	38284	0.94	0.94 – 0.95
	II-	27528	0.92	0.92 – 0.92	10046	0.86	0.85 – 0.86
	II+	50159	0.86	0.86 – 0.86	15529	0.75	0.74 – 0.75
	III	10086	0.61	0.61 – 0.62	2833	0.48	0.47 – 0.49
	IV	2654	0.24	0.23 – 0.24	418	0.12	0.11 – 0.13

Note: *N*_5 _and *N*_10 _correspond to the number of women at risk at the beginning of the interval in the Girona Cancer Registry (GCR) for Catalonia and in the Surveillance Epidemiology and End Results (SEER) for the USA. The 95% confidence intervals were obtained using Greenwood's formula.

### Estimated age- and stage-specific Catalan survival functions

**Figure 5:**
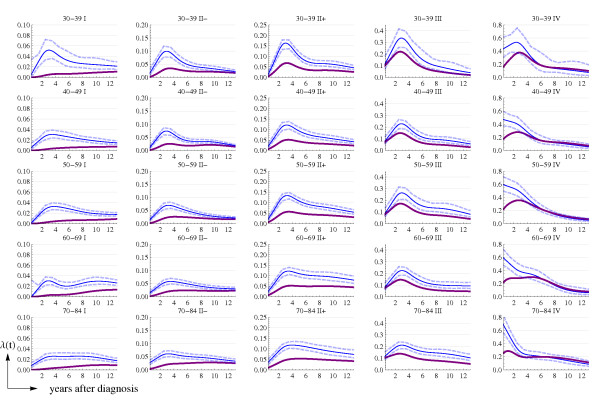
**Estimated age- (years) and stage-specific Catalan hazard rate functions**. Thin line is for the period 1980–89 and thick line for 1990–2001. Dashed lines correspond to the 95% point-wise confidence intervals for the period 1980–89.

**Figure 6:**
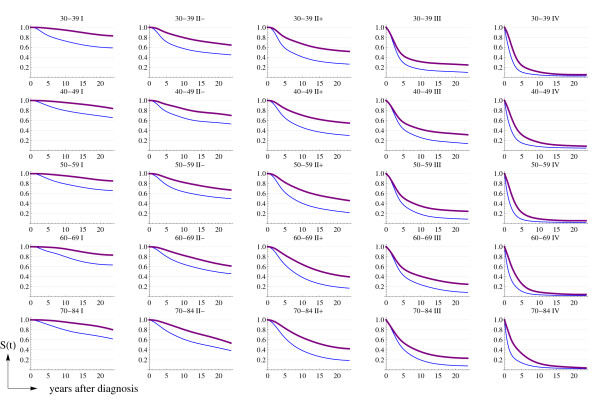
**Estimated age- (years) and stage-specific Catalan cumulative survival**. Thin line is for the period 1980–89 and thick line for 1990–2001.

Figures 5 and 6 display the estimated age- and stage-specific (*t*) and cumulative survival, respectively, for Catalonia in the 1980–89 (thin line) and 1990–2001 (thick line) time periods. These functions have been derived from the  functions in 1975–79 and the corresponding estimated HR. Although Figure 5 shows some overlap between the 95% confidence intervals for the 1980–89 and the 1990–2001 hazards, the estimated functions reflect important improvements in breast cancer survival in Catalonia for all age groups and stages of disease.

## Discussion

Our study achieves two goals. We have obtained age- and stage-specific breast cancer survival functions for Catalonia for two time periods, 1980–89 and 1990–2001. These functions can be used 1) to assess changes over time in survival after breast cancer diagnosis, and 2) to generate breast cancer *pdf *s that are needed to model the impact of early detection and adjuvant treatments in the reduction of breast cancer mortality in Catalonia.

The results of this study show that breast cancer survival in Catalonia during the 1980s was worse than in the USA during the second half of the 1970s. But, during the 1990s breast cancer survival in Catalonia experienced great improvements and ended up being comparable to US survival for most disease stages.

Previous studies compared trends in breast cancer incidence, mortality, and survival in 10 European countries between 1983 and 1994 [22]. Spain and Italy started with the lowest incidence and mortality rates which increased markedly up to the mid 1990s, probably due to changes in lifestyle and diet over time [23]. Despite low mortality, the Spanish relative breast cancer survival in 1984 was among the worst ones according to the EUROCARE study [22]. Fortunately, the increasing trends in breast cancer incidence and mortality (with a maximum around mid 1990s) were accompanied by the steepest increase in relative survival from 1984 to 1994, of all European countries studied, (8% per year) [22]. Whereas breast cancer impact on mortality increased, relative survival improved, probably due to the rapid dissemination of scientific and medical advances.

From mid 1990s to the present, breast cancer mortality in Catalonia shows a decreasing trend [24]. The introduction of early screening and adjuvant treatments may have played an important role, as in most of the western countries [25]. Both cancer control interventions seem to have contributed similarly to the reduction in breast cancer mortality in the USA [2]. In Catalonia, the contribution of each still remains to be evaluated and the results of this study will be used as inputs to the Lee and Zelen model [3]. In this work we have observed that breast cancer survival improved from the 1980s to the 1990s within disease stages. Although earlier time of diagnosis for mammography detected cases (lead time bias) may play a role in the estimated survival distributions for the 1990s, the improvement within disease stages may be related to the introduction of better treatments, too.

When comparing breast cancer survival in different populations it is necessary to take into account that differences in overall mortality and life expectancy influence the cause-specific survival in these populations [13,26,27]. Relative survival and cancer-specific survival provide similar results [14,16]. In relative survival, the total mortality hazard is assumed to consist of the general population mortality hazard and of an excess hazard which is attributable to cancer. In the cause-specific survival, on the other hand, the cause-specific hazard is estimated using the cause of death information only. The cause-specific survival rate is calculated by censoring the survival time related to a non-cancer death. The relative survival analysis is often preferred over the cause-specific survival since the cause of death information in the cancer registries may be unreliable or unavailable. In this study, we have worked with breast cancer relative survival which was obtained considering the life tables of the Catalan population [15].

Since survival data in Catalonia was not available at the population level and estimations of survival functions by age groups and disease stages would have been inaccurate and not reliable, we decided to customize the US survival functions. The limitations of our original data led us to undertake this study. Our assumption was that the Catalan functions had age and stage patterns similar to the US functions, but that levels of risk of breast cancer mortality were the Catalan observed levels. As the number of breast cancer cases in the GCR was small compared to the US SEER cases, instead of using the Cox regression model to compare both geographical areas, we used a Poisson regression model that handled the US as the reference group [28]. The Poisson regression model also made it possible to deal with time varying hazard ratios. We assessed the proportionality of hazards assumption and, when necessary, we included *log*(*time*) as a covariate.

Another limitation intrinsic to the type of data that we used is the noise and fluctuation of hazard rates. To deal with this problem we smoothed the data and weighted it by the number of person-years at risk in each time interval. Figures 1 and 2 show that the adjusted hazard rate functions capture the main trends of the raw data. There are several examples in the literature that combine fitting a spline function to the hazards and obtaining a HR as a function of *time *or *log*(*time*) [29,30].

We assessed the calibration of the Catalan estimated functions using the goodness-of-fit deviance statistic. The test did not reject the null hypothesis in all but four of the cases. The discrepancies can be attributed to the instability of both *D*^*bc *^and *person-years at risk *in the GCR. In general, Figure 4 shows that the observed and predicted values of the Catalan cumulative survival functions are very similar. Therefore, the methods used seem to provide a robust estimate of the breast cancer survival functions in Catalonia.

## Conclusion

In summary, we have combined limited breast cancer survival data from Catalonia (Spain) with more robust data from a bigger population, the USA, to obtain robust survival functions for Catalonia. Our methodology has used standard statistical approaches that can be performed using available software. The results of our study are useful for public health and clinical purposes since they provide information on the progress against breast cancer in Catalonia. In addition, our results provide a comparison of Catalonia and the USA in terms of the prognosis of women diagnosed with breast cancer. Finally we have generated survival probability density functions for Catalonia that will be used in the modeling of the impact of screening mammography and adjuvant treatments.

## Competing interests

The authors declare that they have no competing interests.

## Authors' contributions

MR and EV participated in the conception and design of the study. RMG provided data from Girona Cancer Registry. MMA elaborated age-cohort models for estimating general mortality and incidence and mortality for breast cancer. All authors reviewed and critically revised the original and subsequent manuscript drafts, and approved the final manuscript.

## Appendix

### Estimation of the confidence intervals for the US hazard rates

To obtain the variance and confidence intervals of the smoothed US hazard rates, we proceed as follows:

1. We assumed that the number of breast cancer deaths in a specific time interval [*t*, *t *+ 1) follows a Poisson distribution with mean equal to the smoothed hazard rate in the midpoint of the interval, (*t*), times the observed number of woman-years in the interval, . Then *E*(*t*) = (*t*) is the expected number of deaths in each time interval.

2. For each *t*, *t *= 1,..., *T*, we calculated the Pearson residuals , being *O*_*t *_the observed number of breast cancer deaths.

3. We repeated 1,000 times:

(a) We took a random sample with replacement of the residuals, , *t *= 1,..., *T*, and used these values to create a random sample of count data generated under the model using the expression .

(b) We refited the smoothing spline model using  and the observed woman-years, to create a resampled hazard rate function, (*t*), generated under the model.

4. From the 1,000 replicated hazard rate functions we estimated the variance. The 95% confidence interval limits were obtained using the percentiles 2.5 and 97.5 of the bootstrapped hazard rates.

### Estimation of the number of breast cancer deaths in the GCR

To estimate the *number of breast cancer deaths *at each time interval after breast cancer diagnosis, (), we used data from the GCR and also the *relative breast cancer survival *data obtained using the WAERS web application [15]. Data available from the GCR were the *number of women at risk *at the beginning of each time interval (*N*_*t*_), the *number of all-cause deaths *() and the *number of women lost to follow-up *(*W*_*t*_). The relative breast cancer cumulative survival (*S*^*bc*^(*t*)) provided by the WAERS application made it possible to derive the *conditional probability of dying *from breast cancer, (), at each interval [*t, t *+ 1):(15)

Then, solving the following equation for each interval [*t, t *+ 1):(16)

We estimated the number of breast cancer deaths () as:(17)

### Estimation of the woman-years at risk in the GCR

*Woman-years at risk *for each time interval [*t, t *+ 1) were obtained using the expression *N*_*t *_- 0.5 ( + *W*_*t*_) (see previous section for notation). The implied assumption is that either deaths or losses to follow-up occur uniformly in each time interval. In this formula it is not necessary to distinguish if deaths were from breast cancer or other causes. Both types of deaths contribute similarly subtracting *woman-years at risk*.

The number of *woman-years at risk *in each time interval has been used to estimate the *expected number of breast cancer deaths *(*expected*^*bc*^) in the GCR, if the hazard rates were the US rates, as described in the Methods section.

## Pre-publication history

The pre-publication history for this paper can be accessed here:

http://www.biomedcentral.com/1471-2407/9/98/prepub

## Supplementary Material

Additional file 1 **Number of women diagnosed with breast cancer in the Girona Cancer Registry by age and stage.** The table shows the low number of women diagnosed with breast cancer when stratifying by age and stage. Click here for file
