# Supplementary material for: Estimation of age- and stage-specific Catalan breast cancer survival functions using US and Catalan survival data
Source: BMC Cancer. 2009 Mar 30;9:98. doi: 10.1186/1471-2407-9-98 (PMC2679763; doi:10.1186/1471-2407-9-98)
Supplement: Additional file 1 — Number of women diagnosed with breast cancer in the Girona Cancer Registry by age and stage. The table shows the low number of women diagnosed with breast cancer when stratifying by age and stage. [file 1471-2407-9-98-S1.pdf]

Additional file 1

Number of women diagnosed with breast cancer in the Girona Cancer Registry by age and stage.

Period	Stage	Age (yrs.)					
		≤39	40-49	50-59	60-69	≥70	Total
1980-1989	SEER Historical stage						
	Localized	35	83	117	141	142	518
	Regional	54	117	135	145	167	618
	Distant	5	15	24	38	48	130
1990-2001	AJCC stage						
	I	27	102	103	107	67	406
	II-	27	46	41	55	49	218
	II+	41	90	93	75	68	367
	III	12	35	39	34	49	169
	IV	10	18	13	32	34	107
